# Electrical Insulation and Radar-Wave Absorption Performances of Nanoferrite/Liquid-Silicone-Rubber Composites

**DOI:** 10.3390/ijms231810424

**Published:** 2022-09-09

**Authors:** Wei-Feng Sun, Peng-Bo Sun

**Affiliations:** 1 School of Electrical and Electronic Engineering, Nanyang Technological University, Singapore 639798, Singapore; 2 School of Electrical and Electronic Engineering, Harbin University of Science and Technology, Harbin 150080, China

**Keywords:** strontium ferrite, carbonyl iron, liquid silicone rubber, radar-absorbing material

## Abstract

Novel radar-wave absorption nanocomposites are developed by filling the nanoscaled ferrites of strontium ferroxide (SrFe_12_O_19_) and carbonyl iron (CIP) individually into the highly flexible liquid silicone rubber (LSR) considered as dielectric matrix. Nanofiller dispersivities in SrFe_12_O_19_/LSR and CIP/LSR nanocomposites are characterized by scanning electronic microscopy, and the mechanical properties, electric conductivity, and DC dielectric-breakdown strength are tested to evaluate electrical insulation performances. Radar-wave absorption performances of SrFe_12_O_19_/LSR and CIP/LSR nanocomposites are investigated by measuring electromagnetic response characteristics and radar-wave reflectivity, indicating the high radar-wave absorption is dominantly derived from magnetic losses. Compared with pure LSR, the SrFe_12_O_19_/LSR and CIP/LSR nanocomposites represent acceptable reductions in mechanical tensile and dielectric-breakdown strengths, while rendering a substantial nonlinearity of electric conductivity under high electric fields. SrFe_12_O_19_/LSR nanocomposites provide high radar-wave absorption in the frequency band of 11~18 GHz, achieving a minimum reflection loss of −33 dB at 11 GHz with an effective absorption bandwidth of 10 GHz. In comparison, CIP/LSR nanocomposites realize a minimum reflection loss of −22 dB at 7 GHz and a remarkably larger effective absorption bandwidth of 3.9 GHz in the lower frequency range of 2~8 GHz. Radar-wave transmissions through SrFe_12_O_19_/LSR and CIP/LSR nanocomposites in single- and double-layered structures are analyzed with CST electromagnetic-field simulation software to calculate radar reflectivity for various absorbing-layer thicknesses. Dual-layer absorbing structures are modeled by specifying SrFe_12_O_19_/LSR and CIP/LSR nanocomposites, respectively, as match and loss layers, which are predicted to acquire a significant improvement in radar-wave absorption when the thicknesses of match and loss layers approach 1.75 mm and 0.25 mm, respectively.

## 1. Introduction

Radar stealth technology refers to that the specific surface material can absorb and dissipate incident radar waves to minimize wave reflections when external radar waves enter the surface of military equipment, thus to achieve stealth that radar detection cannot correctly judge the actual size and position of military equipment [1,2]. Radar-wave absorption is mainly realized by coating materials, which can be modified and alternated according to the requirements of the working environment, equipment shape, and absorption rate. Radar-wave absorption coating is flexible, convenient, adjustable and indispensable in radar stealth technologies [3,4]. Wave-absorbing materials constitute a key research topic for scientific researchers and scholars in military and civil research prospects.

According to absorption manners, radar-wave-absorbing materials are classified into absorbent and interferometric categories. Wave-absorbent materials rely on loss and attenuation by absorbing incident radar waves, which are determined by the electromagnetic-loss characteristics of the material itself. Conforming to wave-phase interference principle, the interferometric wave-absorbing materials are exploited to eliminate wave reflection by modulating incident and reflected radar waves into opposite phases, which however can merely be realized for a narrow frequency band. Meanwhile, radar-wave absorbents are further classified into magnetic loss and electric loss types. In particular, electric loss absorbents can be subdivided into electric resistance and dielectric loss classes according to their polarization forms. At present, the widely studied electric resistance wave-absorbing materials include carbon-based composites and conductive polymers [5,6,7,8,9,10]. Dielectric materials are applied in radar-wave absorption through dielectric polarization and relaxation loss. Nowadays, studies on dielectric wave-absorbing materials focus on barium titanate perovskites, barium ferric acid, and ferroelectric ceramics [11,12,13,14]. Magnetic radar-absorbing materials rely on magnetic loss to absorb and dissipate radar electromagnetic waves, which is competent of matching impedance as implemented in composite materials mainly composed of dielectric rubber matrix and functional fillers such as ferrite, carbonyl iron, ultrafine metal powder [15,16,17,18].

Ferrite materials are widely used for radar-wave absorption due to their large-area hystereses of dielectric polarization and magnetization, favoring both magnetic and dielectric losses of incident radar waves. M-type barium ferrite composites doped with cobalt and zirconium show excellent radar-wave absorption performances, approaching a minimum reflection loss of −28.7 dB at 16.4 GHz and an effective absorption bandwidth of 4.46 GHz [19]. Nickel-zirconium/barium-ferrite composites render a minimum reflection loss of −60.6 dB in a broad radar band by film materials of 2.1 mm thickness [20]. Composite ferrite materials made by partially substituting barium ferrite with cobalt ion can acquire a minimum reflective loss of 32.1 dB at 11.2 GHz with a material thickness of 2 mm [21]. The composites of ferrosoferric oxide and stannic oxide in material thickness of 1.7 mm reach a minimum reflective loss of −29 dB and an effective absorption bandwidth of 4.9 GHz [22].

Prospective wave-absorbing materials are now heading towards thin, light, wide, and strong directions, in which radar-wave absorption nanocomposites have mostly been focused in recent years [23,24]. The sizes of nanoparticles filled into radar-absorbing nanocomposites are in 1~100 nm dimension, which favors achieving impedance matching, whilst the atomic absorption of hysteresis loss on nanofiller surfaces can attenuate incident radar waves with an extremely high efficiency [25]. The minimum reflection loss of the mesh-structured nanocomposites prepared by filling 10~70 nm titanium carbide nanoparticles on surfaces of carbon nanotube fibers approaches −45.6 dB at 16.5 GHz, which is even improved to −55.3 dB at 12.8 GHz in molybdenum-disulfide/graphene nanocomposites [26,27]. Carbon-based composites of silicon carbide nanowires of 20~60 nm diameter coated with graphene achieve a minimum reflection loss of −16.2 dB and an effective absorption bandwidth of 2.64 GHz [28]. Microwire composites have been demonstrated to be flexibly manipulated for modifying microwave response by magnetic field/stress stimuli or hybridization and by assembling wire functional units in a specific arrangement, which expands the applications of fiber-reinforced composites in fields such as information, energy, and security technologies [29].

Liquid silicone rubber (LSR) has been comprehensively used as a matrix material for preparing composite materials due to its high levels of tear resistance strength, thermal stability, process flexibility, and mechanical elasticity, which also favors acquiring high dispersivity of inorganic nanofillers in rubber-based wave-absorbing composites. Nanoscaled ferrite and iron powders are high-efficiency magnetic loss fillers for preparing radar-absorbing polymer dielectric composites. Strontium ferrite (SrFe_12_O_19_) is a classical M-type hexagonal ferrite with a hard magnetism, and has been comprehensively studied for applications in microwave devices [30,31]. Nano-SrFe_12_O_19_ also possesses the above-mentioned features of M-type ferrites. Carbonyl iron (CIP) nanopowder represents high dielectric constant, magnetic loss, and permeability, which can withstand a relatively high Curie temperature and exhibits excellent wave absorption performances in low-frequency microwave bands [4]. It has been basically established that nanoscale SrFe_12_O_19_ or CIP and LSR render favorable characteristics of magnetic fillers and dielectric matrix, respectively, for realizing high-performance radar-absorbing composites. However, there is still no report on these sorts of composite materials for acquiring higher radar-wave absorption performances.

In order to reduce electromagnetic wave pollution and advance radar stealth technology, the present study utilizes the addition molding two-component (AMT) liquid silicone rubber (LSR) as dielectric matrix and strontium ferrite (SrFe_12_O_19_) or carbonyl iron (CIP) nanoparticles as fillers to develop SrFe_12_O_19_/LSR and CIP/LSR nanocomposites with high performance in electric insulation and radar wave absorption, which are elucidated by characterizing nanofiller dispersion and testing the mechanical tensile strength, electric conductivity, direct-current (DC) dielectric-breakdown strength, electromagnetic-response characteristics, and radar-wave reflectivity. Electromagnetic transmission simulations are performed to evaluate radar-wave scattering of dual-layer structures constituted by SrFe_12_O_19_/LSR and CIP/LSR nanocomposites, which are dedicated to the optimization of material type and loss layer thickness for improving radar-wave absorption.

## 2. Results and Discussion

### 2.1. Micromorphology Characterization

**Figure 1:**
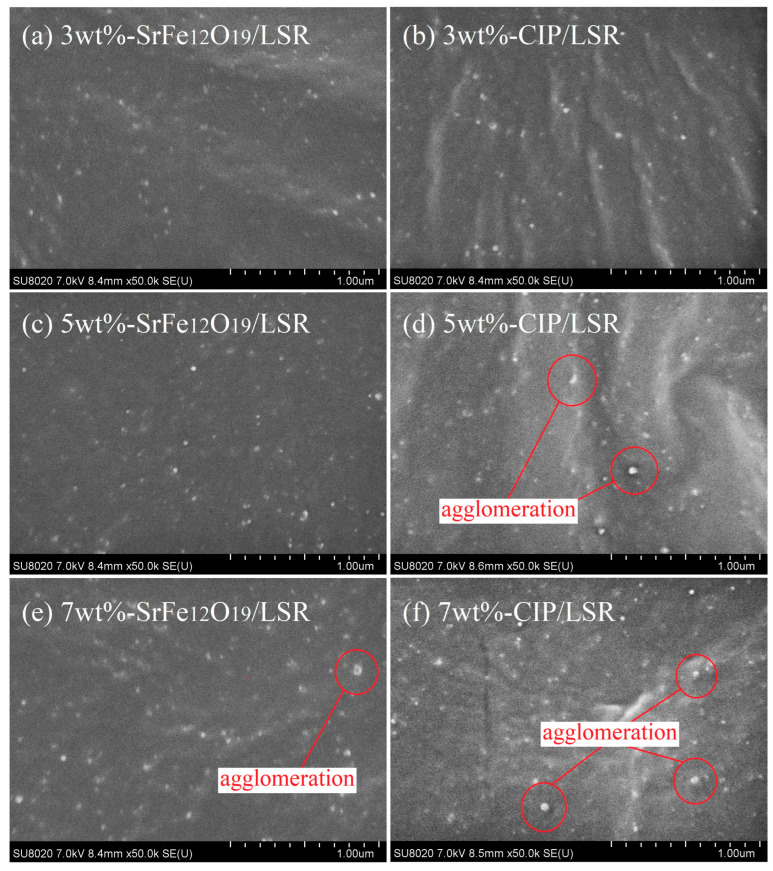
Cross-sectional SEM images of SrFe_12_O_19_/LSR and CIP/LSR nanocomposites.

As illustrated by cross-sectional SEM images in Figure 1, the SrFe_12_O_19_/LSR and CIP/LSR nanocomposites show a highly uniform distribution with a high dispersivity but with different-sized nanofillers in LSR matrix. With the increase in filling content, the nanofiller size increases due to the pristine filler agglomerations, as especially manifested in 7 wt%-CIP/LSR nanocomposite. In contrast, the 7 wt%-SrFe_12_O_19_/LSR nanocomposite represents a preferable trend of mitigatory filler agglomeration with smaller filler sizes.

It is noted that the mechanical strength and insulation performance of the prepared composites should be necessarily considered for practical applications of radar absorption coatings, so the use of a matrix medium with some special dielectric polymers for realizing actual radar-absorbing composites is preferred. Therefore, we adopt LSR, which has comprehensive advantages of high heat stability, high electric resistance, ease of processing, etc. to act as the consistent dielectric matrix supportting functional ferrite nanofillers. Even though the filling content is below 7% for acquiring a considerable radar absorption (as shown in the following sections), filling the magnetic nanofillers into LSR will lead to the degradations of mechanical strength and insulation performance due to the inhomogeneity caused by filler aggregations (as shown in Figure 1f). Therefore, in the following two sections, the mechanical tensile strength, electric conductivity, and dielectric-breakdown strength are tested to evaluate the feasibility of applying these LSR nanocomposites in dielectric coating materials before elucidating radar-wave response and reflection characteristics.

### 2.2. Mechanical Tensile Properties

**Figure 2:**
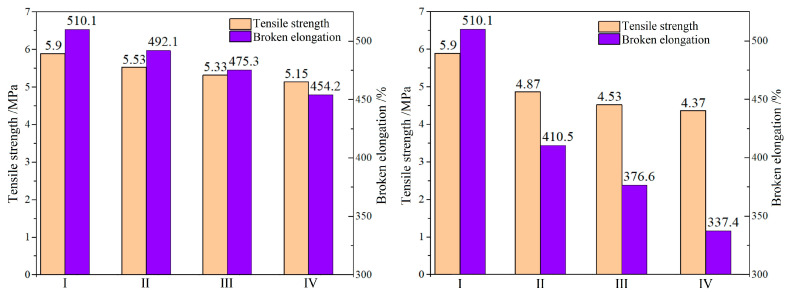
Tensile strength and broken elongation of SrFe_12_O_19_/LSR (**left panel**) and CIP/LSR (**right panel**) nanocomposites where I, II, III, and IV denote the filling contents of 0, 3, 5, and 7 wt%.

Mechanical tensile properties of SrFe_12_O_19_/LSR nanocomposites are slightly lower than that of LSR, as shown in Figure 2. Compared with LSR, the SrFe_12_O_19_/LSR nanocomposites with filling contents of 3 wt%, 5 wt% and 7 wt% present the tensile strengths lower by 6.3%, 9.7% and 12.7% respectively, and broken elongations lower by 3.5%, 6.8% and 11.0% respectively. In contrast, the filling content has a relatively obvious effect on mechanical tensile properties of CIP/LSR nanocomposites, as shown in the right panel of Figure 2. With reference to LSR, the tensile strengths of CIP/LSR nanocomposites with filling contents of 3 wt%, 5 wt% and 7 wt% decrease by 17.4%, 23.2% and 25.9% respectively, whilst their broken elongations are reduced by 19.3%, 26.2% and 33.8% respectively. In comparison with SrFe_12_O_19_ nanofillers, the inorganic magnetic CIP nanofillers are less compatible with organic LSR dielectric matrix, leading to the lower composite intensity, as consistently indicated by the observable filler agglomeration of 7 wt%-CIP/LSR nanocomposite in SEM Figure 1f, which causes macroscopic strains in a larger area around CIP nanofillers, accounting for the lower mechanical tensile properties of CIP/LSR nanocomposites than those of SrFe_12_O_19_/LSR nanocomposites.

### 2.3. Electrical Insulation Performance

**Figure 3:**
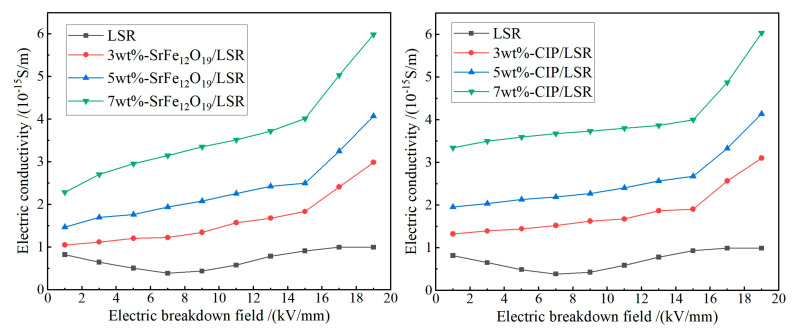
Electric conductance *γ*-*E* curves of SrFe_12_O_19_/LSR (**left panel**) and CIP/LSR (**right panel**) nanocomposites.

Electric conductivities of both SrFe_12_O_19_/LSR and CIP/LSR nanocomposites are somewhat higher than that of LSR due to the filler-introduced electronic-states near band-edge in the bandgap of LSR which contribute charge carriers under thermal excitation at room temperature, as indicated by the profiles of electric conductivity versus electric field strength (*γ*-*E* curves) in Figure 3. The higher filling content leads to a higher electric conductivity for both SrFe_12_O_19_/LSR and CIP/LSR nanocomposites, such as 7 wt% filling content results in twice the conductivity of 3 wt% filling content, while the LSR conductivity remains nearly unchanged over the measured range of electric field strengths. Both SrFe_12_O_19_/LSR and CIP/LSR nanocomposites exhibit a linear *γ*-*E* curve under a low electric field, which however represents a significant nonlinearity under the electric field higher than 15 kV/mm, especially for the filling content higher than 5 wt%.

Most of the charge carriers dedicated to electric conductance are in the localized states residing at filler/matrix interfaces in polymer dielectric nanocomposites (nanodielectrics). The process of charge transporting between these localized states is described macroscopically as percolation conductance, which will be exponentially expedited by thermal excitation, as characterized by the conductivity nonlinearity arising in the nanodielectrics with a high filler concentration. According to percolation conductance theory [32], the polarized interface layer around SrFe_12_O_19_ or CIP nanofillers will overlap to form a random conductive network when the concentration of nanofillers in LSR matrix exceeds a percolation threshold, resulting in the percolation conductance which accounts for the nonlinear conductivity of SrFe_12_O_19_/LSR and CIP/LSR nanocomposites [33]. In addition, the lower compatibility and larger size of nanofiller result in more filler-introduced charge carriers and a greater percolation conductance, respectively, accounting for the higher conductivity of CIP/LSR nanocomposites than that of SrFe_12_O_19_/LSR nanocomposites.

**Figure 4:**
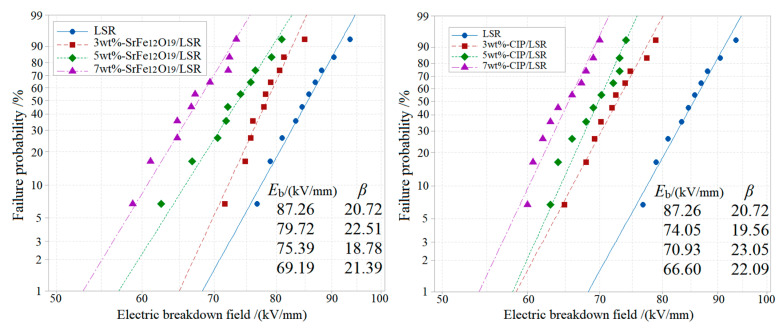
Weibull distributions of electric breakdown field strength of SrFe_12_O_19_/LSR (**left panel**) and CIP/LSR (**right panel**) nanocomposites, in which *E*_b_ and *β* denote characteristic breakdown field and shape parameter, respectively.

The DC breakdown field strengths are analyzed by Weibull statistics, as shown in Figure 4, implying the lower dielectric-breakdown strength (as described by the characteristic breakdown field *E*_b_) of both nanocomposites than that of LSR, which further abates with increasing filling content. Compared with LSR, the SrFe_12_O_19_/LSR nanocomposites with filling contents of 3, 5 and 7 wt% decline by 8.6%, 13.6% and 20.7% respectively in characteristic breakdown field, while CIP/LSR nanocomposites with these filling contents fall by larger magnitudes of 15.14%, 18.71% and 23.68% respectively. Since SrFe_12_O_19_ or CIP nanofillers introduce charge carriers and even cause percolation conductance under high electric fields and high filling contents, which is more evident for CIP nanofillers as shown by electric conductivity in Figure 3, the breakdown resistances of their nanocomposites are inevitably decreased in comparison with LSR. Therefore, the insulation persistence or degradation of LSR nanocomposites is highly reliant on the mixing compatibility of magnetic inorganic nanofillers with LSR matrix, as indicated by the appreciably higher insulation performances of SrFe_12_O_19_/LSR nanocomposites than that of CIP/LSR nanocomposites. 

### 2.4. Electromagnetic-Response Characteristics

Reflected radar waves mainly consist of two parts: one part is derived from surface reflection on material surface before incidence into material; the other part is contributed by the attenuated radar waves transmitting out of the internal material. Thus, it is a merit to inhibit radar reflections by improving internal loss factor as well as by matching impedance.

**Figure 5:**
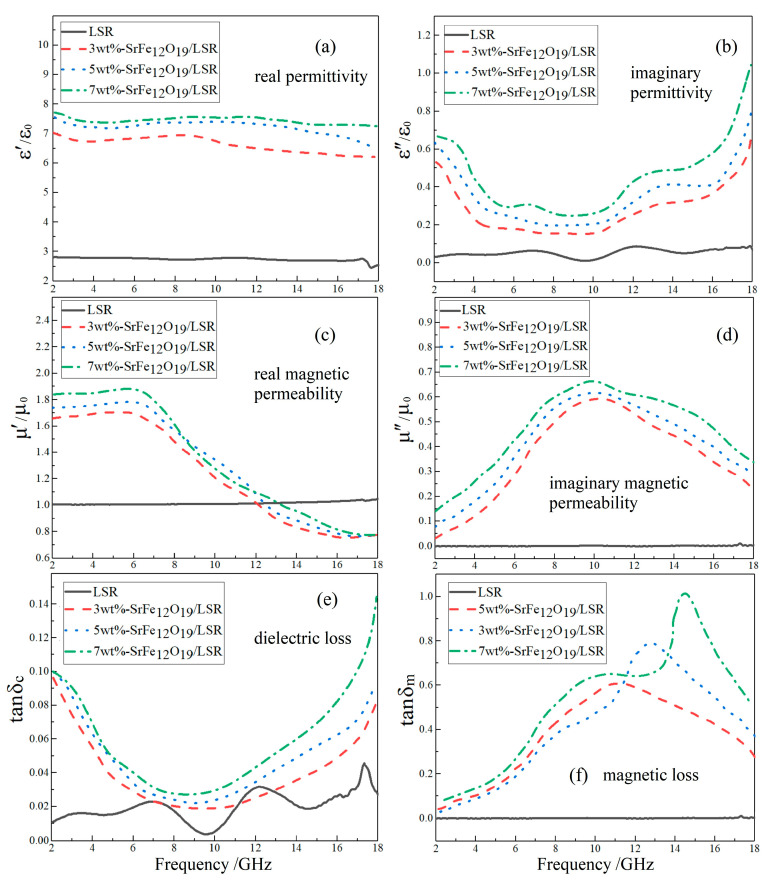
Electromagnetic-response frequency spectra of SrFe_12_O_19_/LSR nanocomposites: (**a**–**d**) complex dielectric permittivity and magnetic permeability; (**e**,**f**) dielectric and magnetic losses.

In the radar frequency band of 2~18 GHz, SrFe_12_O_19_/LSR nanocomposites give a real dielectric permittivity (*ε*′/*ε*_0_) of 6~8 with a slight dependence on frequency, which is almost proportional to filling content in contrast to the constant 2.8 of LSR, as shown in Figure 5a. The imaginary dielectric permittivity (*ε*″/*ε*_0_) in the frequency response spectra of SrFe_12_O_19_/LSR nanocomposites, which rises slightly with increasing filling content, resides in the range of 0.2~1, as shown in Figure 5b. In contrast to constant value of 1.0 for LSR, the real part of magnetic permeability (*μ*′/*μ*_0_) for SrFe_12_O_19_/LSR nanocomposites varies evidently with frequency, approaching the maximum value of 1.7~1.9 at 6 GHz, as shown in Figure 5c. Hence, it is preferential to reach impedance matching under the lower frequency region due to the smaller difference between *μ*′ and *ε*′. The imaginary part of magnetic permeability (*μ*″/*μ*_0_) is lower than 0.7 of the highest value at 10 GHz in contrast to the nearly zero value of LSR, as shown in Figure 5d.

To intuitively evaluate loss factors, the dielectric and magnetic losses are calculated from the complex dielectric permittivity and the complex magnetic permeability, as shown by the results in Figure 5e,f. SrFe_12_O_19_ is a kind of wave-absorbing material with both dielectric and magnetic losses, while LSR matrix only has dielectric loss. The dielectric losses (tan*δ*_c_) of SrFe_12_O_19_/LSR nanocomposites reside in the 0.03~0.15 range with the minimum value arising at 8~10 GHz, which could be entirely promoted by increasing filling content. In contrast, the magnetic loss (tan*δ*_m_) is much greater, for example reaching 1.0 at 15 GHz for 7 wt%-SrFe_12_O_19_/LSR nanocomposite, implying that the absorption of incident radar waves is dominated by magnetic loss. It is demonstrated that SrFe_12_O_19_/LSR nanocomposites favor radar-wave absorption from magnetic losses in the high-frequency region of radar band.

**Figure 6:**
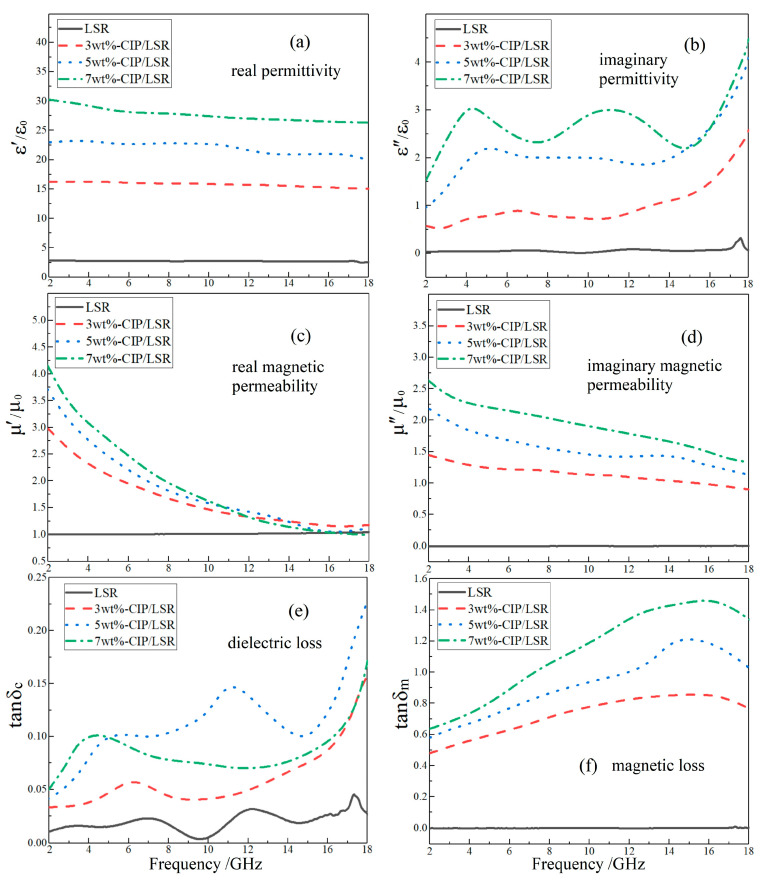
Electromagnetic-response frequency spectra of CIP/LSR nanocomposites: (**a**–**d**) complex dielectric permittivity and magnetic permeability; (**e**,**f**) dielectric and magnetic losses.

Electromagnetic parameters of CIP/LSR nanocomposites are significantly higher compared to LSR in the whole tested band of 2~18 GHz, which increase with increasing filling content, as shown in Figure 6. The *ε*′ of CIP/LSR almost remains independent of frequency, while the smallest value of 3 wt%-CIP/LSR persists higher than 15. By contrast, the *ε*″ fluctuates with frequency, acquiring the highest value of >4 for 7 wt%-CIP/LSR. However, the complex magnetic permeability (*μ*′ and *μ*″) declines with increasing frequency, as manifested by 7 wt%-CIP/LSR whose *μ*′ decreases from 4.2 to 1.0 and *μ*″ decreases from 2.6 to 1.5. Therefore, it is preferable for CIP/LSR nanocomposites to fulfill impedance matching in the low-frequency region where the difference between *ε*′ and *μ*′ is minimized.

Dielectric loss peaks of CIP/LSR nanocomposites depend greatly on filler content, whilst the entire magnitude of magnetic loss rises up as the filling content is raised. Magnetic losses increase monotonously with increasing frequency, approaching the maximum value of 1.5 at 14 GHz for 7 wt%-CIP/LSR nanocomposite. In the whole tested band of 2~18 GHz, the magnetic losses are much higher than dielectric losses, verifying that radar-wave absorption is dominantly derived from magnetic losses in CIP/LSR nanocomposites, with a greater minor contribution from conductance losses due to the considerably higher conductivity than that of SrFe_12_O_19_/LSR nanocomposites.

### 2.5. Radar Reflections

**Figure 7:**
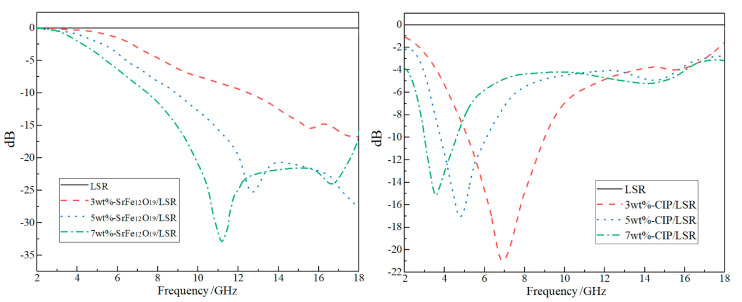
Radar-wave reflectivity of SrFe_12_O_19_/LSR (**left**) and CIP/LSR (**right**) nanocomposites.

Radar reflectivity frequency spectra and radar absorption characteristics (minimum reflection loss and effective absorption bandwidth) are respectively shown in Figure 7 and Table 1. Absorption peaks of SrFe_12_O_19_/LSR nanocomposites are mainly concentrated in high-frequency region, in which the peak position shifts towards a lower frequency from 15.2 to 11.0 GHz when the filling content is raised from 3 wt% to 7 wt%. Meanwhile, both the minimum reflection loss (MRL) and effective absorption bandwidth (*f*_E_) of SrFe_12_O_19_/LSR can be evidently improved by raising filling content, as indicated in Table 1. In contrast, the absorption peaks of CIP/LSR concentrate in low-frequency region, with both MRL and *f*_E_ decreasing with the increase of filling content. Meanwhile, the *f*_E_ values of CIP/LSR are remarkably smaller than those of SrFe_12_O_19_/LSR. Furthermore, the peaking point of CIP/LSR shifts towards a lower frequency from 7.0 GHz to 3.7 GHz as the filling content increases from 3 wt% to 7 wt%. In the right columns of Table 1, the radar-absorbing performances of ferrite-ceramic and carbon-based composites from recent reports are also listed in comparison to the present research. It suggests that SrFe_12_O_19_/LSR nanocomposites reach the advanced radar absorption performances, and even 7 wt%-SrFe_12_O_19_/LSR nanocomposite resides on the top level, as comprehensively evaluated by MRL, *f*_E_ and absorbing-film thickness.

Employing the melting and mixing method is limited by the compatibility between filler and matrix materials, as manifested by the notable agglomeration of CIP/LSR when the filling content approaches 7 wt% (as shown in Figure 1f), which acts against persisting high magnetic loss and insulation strength (as shown in the right panels of Figure 3 and Figure 4, and in Figure 6f). In contrast, with the increase in filling content, the mechanical tensile strength of SrFe_12_O_19_/LSR decreases very little whilst persisting the increase in radar absorption performance. On the contrary, the radar absorption performances of CIP/LSR decrease with filling content, as shown in Table 1. Therefore, for applying the CIP/LSR nanocomposites to a single- or double-layer structure of radar-wave absorption coating, the filling content should be controlled below 5%, but the filling content of SrFe_12_O_19_/LSR is preferred to be higher (7w% or more) for obtaining higher radar-wave absorption performances while maintaining adequate mechanical and insulation strength.

### 2.6. Simulations of Dual-Layer Radar Reflection

Electromagnetic-response and radar-reflection characteristics indicate that: SrFe_12_O_19_/LSR nanocomposites can provide a high reflection loss under high filler concentrations in favor of high-frequency radar-wave absorption, but show a low reflection loss with a narrow *f*_E_ under low filler concentrations; CIP/LSR nanocomposites have excellent wave absorption performances but a narrow *f*_E_ in low-frequency region of radar band. In practical engineering applications, only a single layer of wave absorption coating cannot balance the absorption frequency band, effective absorption bandwidth, and reflection loss.

**Figure 8:**
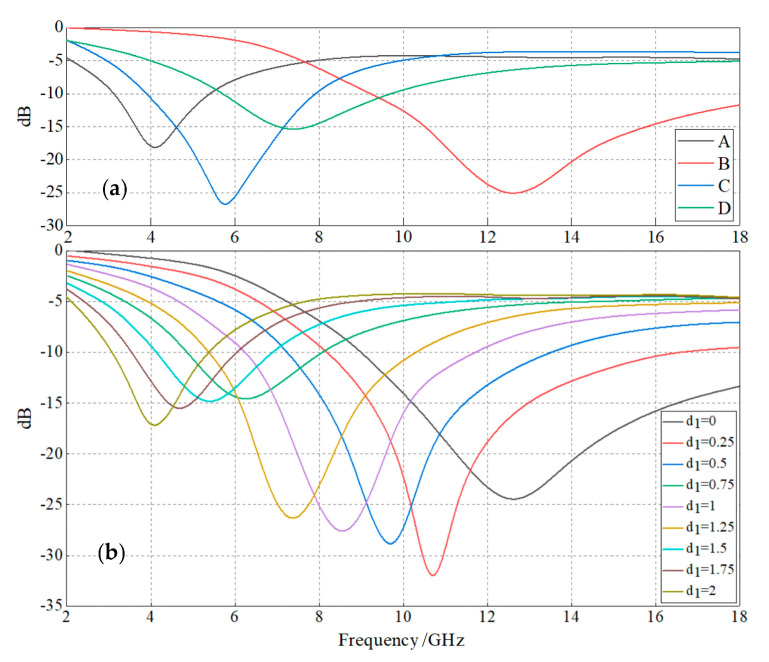
Radar reflectivity frequency spectra from electromagnetic wave transmission simulations: (**a**) single-layer and dual-layer structures comprised of SrFe_12_O_19_/LSR or CIP/LSR nanocomposites; (**b**) dual-layer structure comprised of SrFe_12_O_19_/LSR nanocomposite match layer and CIP/LSR nanocomposite loss layer for various loss layer thicknesses. Radar-wave incident (electromagnetic transmission) direction is specified as perpendicular to layer plane.

The 5 wt%-SrFe_12_O_19_/LSR and 5 wt%-CIP/LSR nanocomposites are individually specified as the match layer or loss layer according to their electromagnetic-response properties for constituting dual-layer structures with 5 × 5 mm^2^ area and 2 mm total thickness. Four kinds of coatings are simulated for radar reflection characteristics: (A) CIP/LSR single-layer, (B) SrFe_12_O_19_/LSR single-layer; (C) dual-layer with SrFe_12_O_19_/LSR and CIP/LSR as match and loss layers respectively; (D) dual-layer with CIP/LSR and SrFe_12_O_19_/LSR as match and loss layers respectively. Material type and thickness of individual layers are altered for minimizing radar reflectivity, as shown in Figure 8a.

It is noted comparatively that C renders the highest wave absorption and D gives the greatest reflection loss. Due to the large discrepancy between the real dielectric permittivities of CIP/LSR nanocomposite and air medium, the CIP/LSR match layer in the D structure cannot realize favorable impedance matching with air medium, leading to a low radar-wave incidence into loss layer, which accounts for the higher reflectivity. By contrast, the SrFe_12_O_19_/LSR as match layer in C structure presents a similar real dielectric permittivity as air medium to successfully fulfill impedance matching for introducing radar waves into loss layer, resulting in the significantly lower reflectivity. In addition, the C and D structures give the absorption peaks located at the frequencies between the MRL frequencies of A and B single layers, whilst rendering a higher *f*_E_ than CIP/LSR single-layer (A). It is hereby suggested for practical applications that the radar-absorbing coating is designed according to the operation frequency, the expected *f*_E_, and the required reflectivity.

Specifically for C dual-layer with a constant total thickness of 2 mm, the loss layer thickness is set as a variable *d*_1_ in the range of 0~2 mm, as shown in Figure 8b. With the increase in loss layer thickness, the minimum reflection loss and the magnitude of the absorption peak increase in the ranges of 1.25~2 mm and 0.25~1 mm, respectively. When CIP/LSR loss layer approaches the thickness of 0.25 mm (SrFe_12_O_19_/LSR match layer approaches 1.75 mm thickness), the highest wave absorption performance is acquired by reaching the minimum reflection loss of −33 dB. Whereas, the wave absorption performances of dual layers will be lower than that of single-layers when loss layer is in thicknesses of 1.25~2 mm. Accordingly, it is suggested for designing dual-layer coatings to simultaneously consider the thicknesses of both loss and match layers without fixing the total thickness.

## 3. Materials and Methods

### 3.1. Material Preparations

Raw materials for preparing SrFe_12_O_19_/LSR and CIP/LSR nanocomposites are listed in Table 2, and the fundamental properties of addition molding two-component (AMT) LSR after vulcanization are also listed in Table 3 for reference. Nanodielectrics have been comprehensively developed for improving electrical performances or acquiring specific features from nanofillers, which employs a small content of inorganic nanoparticles (such as nanoscaled materials of aluminum oxide, nanosilica, and bismuth ferric acid) filled into dielectric polymers of silicone rubber or crosslinked polyethylene [34,35,36]. Based on the previous researches on nanodielectrics, it is preferable to use inorganic nanoparticles in sizes of 20~60 nm for preparing polymer-matrix nanocomposites with the melting blend method, in which the filling content of inorganic nanoparticles is generally lower than 5 wt% to adequately avoid agglomerations of nanofillers [37,38,39].

The equal-quality LSR raw materials of component A (vinyl-sealing silicone oil and catalyst platinum) and component B (polymethylvinyl siloxane and crosslinker containing hydrosilicone oil) are firstly blended in an iron beaker with a multifunctional dispersion mixer until the two components A and B are evenly mixed without any observable stratification. Secondly, the nanoscaled material of SrFe_12_O_19_ or CIP is added into the two-component LSR mixture to be stirred evenly, and then put into vacuum-drying oven to remove the air bubbles from the mixture. Thirdly, the obtained mixture is heated up to 120 °C at a heat-rate of 5 °C/min and boosted to 15 MPa at a press-rate of 1 MPa/ min in a plate vulcanizer, persisting for 30 min to realize the first crosslinking process of LSR matrix and the composite process of nanofillers with matrix. Eventually, the prepared composite materials are hot-degassed at 200 °C for 4 h in an electrothermal air-blast cabinet to fulfill the second crosslinking reinforcement, which is applied for improving mechanical properties. Through this method, the SrFe_12_O_19_/LSR and CIP/LSR nanocomposites with filling contents of 3, 5 and 7 wt% are produced. Experimental instruments for material preparations are listed in Table 4.

### 3.2. Material Characterization and Performance Testing Methods

Cross-sectional micromorphology of nanocomposite materials is characterized with scanning electronic microscope (SEM, SU8020, Hitachi Hi-tech Group, Tokyo, Japan) to evaluate the dispersivity of nanofillers. The film material of 0.75 mm thickness is placed into liquid nitrogen and promptly taken out to be cold brittle broken, the cross-section of which is sprayed with a silver film for SEM observation.

Stress–strain characteristics are tested, conforming to the GB/T 1040.2-2006 standard, by specifying the elongation speed of 5 mm/min and the testing temperature of 25 °C, in which the material specimen is shaped into a “5A” dumbbell of 6mm width and 0.75 mm thickness with a mark distance of 20 mm.

Electric conductance is analyzed from the steady-state conductance current (or transformed to conductivity) as a function of the increasing electric field in step boost mode in the range of 1~20 kV/mm at ambient temperature, which is tested with the three-electrode method and recorded for each test point after applying voltage for 60 min. The film material specimen of 0.2 mm thickness is evaporated by an aluminum electrode as high-voltage electrode on one side and by two aluminum electrodes respectively for measuring and protection on the other side. Direct-current (DC) electric breakdown field strength is tested by recording the maximum voltage just before the specimen undergoes a dielectric breakdown when the applied voltage is raised at a constant rate of 2 kV/s, for which the circular film specimen of 0.2 mm thickness is evaporated by asymmetric columnar electrodes of diameters 25 mm and 75 mm for high-voltage and ground electrodes, respectively. In order to avoid surface creepage discharges, the breakdown test specimen and the whole electrode system are immersed in insulating silicone oil when applying voltage.

**Figure 9:**
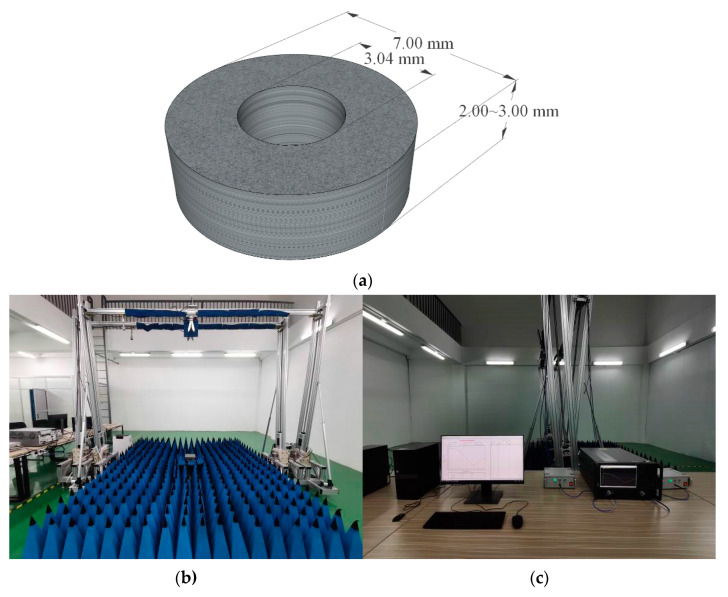
(**a**) Schematic sample shape and sizes for testing electromagnetic-response characteristics through the coaxial method, and photo images of (**b**) test system and (**c**) segmental support for testing radar reflectivity.

Electromagnetic-response characteristics, which determine the radar-absorbing performances, are tested on the hollow cylindrical specimen of thickness 2~3 mm with a 7 mm outer diameter and 3.04 mm inner diameter (as shown in Figure 9) using a microwave network analyzer (Keysight N5224B, Tekolaire Co. Ltd., Beijing, China) in the frequency range of 2~18 GHz. Radar reflectivity is tested complying with the GJB 2038A-2011 standard, as implemented by the test system shown in Figure 9b,c, in which the film specimen is required in a square shape, with the size being modified according to the testing frequency range, as shown in Table 5.

### 3.3. Simulation Method of Electromagnetic Transmission

Radar-wave transmissions through nanocomposite layer structures are analyzed with three-dimensional electromagnetic-wave simulation software CST Microwave Studio according to the experimental electromagnetic-response characteristics of nanocomposites, in which the radar-absorbing material and layer thickness in dual-layer structures are investigated for minimizing radar reflectivity. CST Microwave Studio provides comprehensive algorithms for time and frequency domains in a variety of electromagnetic frequency ranges, which can be employed in researches on communication antenna, radar-wave absorption, intelligent cars, mobile phones, and high-power microwaves. For dual-layer wave-absorbing models, the plate metal with a perfect electric conductance is applied as the layer substrate on which the 5 × 5 × 2 mm^3^ cubic layers with electromagnetic parameters of SrFe_12_O_19_/LSR or CIP/LSR nanocomposites are closely attached to form the wave-absorbing coating.

## 4. Conclusions

Employing nanoscaled strontium ferrite (SrFe_12_O_19_) and carbonyl iron (CIP) as composite magnetic fillers, and liquid silicone rubber (LSR) as dielectric matrix, we developed SrFe_12_O_19_/LSR and CIP/LSR nanocomposites for radar-wave absorption. Electric insulation and radar-absorbing performances are elucidated and analyzed by testing mechanical tensile properties, electric conductivity, DC dielectric-breakdown strength, dielectric and magnetic losses, and radar reflectivity. According to experimental electromagnetic-response characteristics, the single-layer and dual-layer wave-absorbing structures are modeled for simulating electromagnetic-wave transmissions, as implemented in CST Microwave Studio, by which dual-layer structures are modified to achieve the minimized radar reflectivity. 

Both SrFe_12_O_19_/LSR and CIP/LSR nanocomposites show slight reductions in mechanical tensile and dielectric-breakdown strengths as compared to LSR benchmark. Electric conductivity is directly proportional to nanofiller concentration and a significant *γ*-*E* nonlinearity arises under high electric fields due to the filler-introduced percolation conductance. Radar-wave absorption is dominantly derived from magnetic losses; SrFe_12_O_19_/LSR nanocomposites give radar absorption peaks in the high-frequency region of 10~18GHz, reaching a minimum reflection loss of −33 dB at 11 GHz and an effective absorption bandwidth of 10.1 GHz at 7 wt% filling content; CIP/LSR nanocomposites render absorption peaks in the low-frequency region of 2~8 GHz, acquiring the highest radar-absorbing performances at 3 wt% filling content, with a minimum reflection loss of −21 dB at 7 GHz and an effective absorption bandwidth of 3.9 GHz.

Dual-layer wave-absorbing structures are modeled to simulate electromagnetic scattering, demonstrating that a higher radar-wave absorption can be acquired by specifying SrFe_12_O_19_/LSR and CIP/LSR as match and loss layers respectively, while a higher radar reflectivity can be obtained by specifying them as loss and match layers respectively. In particular, the highest wave absorption performances can be reached when tCIP/LSR loss layer is specified at 0.25 mm thickness. The radar reflectance of dual-layer structures depends on the type and thickness of individual constituent materials, which signifies improving radar absorption performances by optimizing multilayer structures.

## Figures and Tables

**Table 1:** Minimum refection loss (MRL) and effective absorption bandwidth *f*_E_ (<−10 dB) in 2~18 GHz range for SrFe_12_O_19_/LSR and CIP/LSR nanocomposites compared with other recent reports.

Nanocomposites/2.0 mm (Present Research)	MRL/−dB	*f*_E_/GHz (<−10 dB)	Nanocomposites (Other Reports)	MRL/−dB	*f*_E_/GHz (<−10 dB)
3 wt%-SrFe_12_O_19_/LSR	14	5.2	[16] CoFe_2_O_4_/NiFe_2_O_4_ nanocapsules/4.5 mm	20.1	8.4
5 wt%-SrFe_12_O_19_/LSR	28	8.5	[19] Co^2+^-Zr^4+^-doped barium ferrite/1.7 mm	28.7	4.5
7 wt%-SrFe_12_O_19_/LSR	33	10.1	[21] BaFe_1__1.6_Co_0.4_O_19_/2.0 mm	32.1	5.0
3 wt%-CIP/LSR	21	3.9	[22] Fe_3_O_4_@SnO_2_ core–shell/1.7 mm	29.0	4.9
5 wt%-CIP/LSR	17	2.3	[28] SiC-NWs@graphene/2.5 mm	16.2	2.6
7 wt%-CIP/LSR	15	1.5	[5] 3D h-BNNS/CNTs/2.5 mm	36.5	4.0

**Table 2:** Raw materials of preparing nanocomposites.

Raw Materials	Model or Particle Diameter	Manufacturer
Addition molding two-component LSR	POWERSIL^®^737	Wacker Chemicals Co., Ltd., Shanghai, China
Strontium ferrite (SrFe_12_O_19_) nanoparticles	100 nm	Hi-tech New Material Technology Co., Ltd., Beijing, China
Carbonyl iron (CIP) nanoparticles	40 nm	Yaotian New Materials Technology Co., Ltd., Shanghai, China

**Table 3:** Fundamental properties of AMT LSR after vulcanization.

Property	Testing Standard	Unit	Value
Density	ISO2781	[g/cm^2^]	1.08
Hardness	ISO868	−	38
Anti-tear strength	ASTM624B	[N/mm]	26

**Table 4:** Experimental instruments for material preparations.

Instrument	Model	Manufacturer
Electronic balance	JE502	Puchun Measurement Instrument Co., Ltd., Shanghai, China
Plate vulcanizer	XLB25-D	Shuangli Automation Technology Equipment Co., Ltd., Huzhou, China
Vacuum-drying oven	DK-150	Sude Test Equipment Co., Ltd., Wuxi, China
Electric mixer	JJ-1	White Pagoda Xinbao Instrument Factory, Jintan, China
Electrothermal air-blast cabinet	101-2AB	Tester Instruments Co., Ltd., Tianjin, China
Multifunctional dispersion mixer	MXD-E1100	Mu Xuan Industrial Co., Ltd., Shanghai, China

**Table 5:** Specimen dimensions for different test frequency ranges.

Specimen Dimension	Frequency Range /GHz
600 mm × 600 mm	0.5~6.0
500 mm × 500 mm	1.0~8.0
300 mm × 300 mm	2.0~18.0

## Data Availability

Theoretical and experimental methods and results are available from all authors.

## References

[1] KAnuradha S., Balakrishnan J. (2021). Resonance Based Discrimination of Stealth Targets Coated with Radar Absorbing Material (RAM). Prog. Electromagn. Res. M.

[2] Jeong H., Nguyen T.T., Lim S. (2018). Meta-Dome for Broadband Radar Absorbing Structure. Sci. Rep..

[3] Kumar A., Singh S. (2018). Development of Coatings for Radar Absorbing Materials at X-Band. IOP Conf. Ser. Mater. Sci. Eng..

[4] Chen L., Gu Z.Z., Zhang M.X. (2018). Microwave Absorbing Property of Thin Coating in the Broadband Low-Frequency range. Mater. Sci. Forum.

[5] Zhong B., Cheng Y.J., Wang M., Bai Y.Q., Huang X.X., Yu Y.L., Wang H.T., Wen G.W. (2018). Three Dimensional Hexagonal Boron Nitride Nanosheet/Carbon Nanotube Composites with Light Weight and Enhanced Microwave Absorption Performance. Compos. Part A Appl. Sci. Manuf..

[6] Silva V.A., Rezende C. (2020). S-parameters, Electrical Permittivity, and Absorbing Energy Measurements of Carbon Nanotubes-Based Composites in X-band. J. Appl. Polym. Sci..

[7] Zhang S.L., Qi Z.W., Zhao Y., Jiao Q.Z., Ni X., Wang Y.J., Chang Y., Ding C. (2017). Core/Shell Structured Composites of Hollow Spherical CoFe_2_O_4_ and CNTs as Absorbing Materials. J. Alloy. Compd..

[8] Fan Z.M., Wang D.L., Yuan Y., Wang Y.S., Cheng Z.J., Liu Y.Y., Xie Z.M. (2020). A Lightweight and Conductive MXene/Graphene Hybrid Foam for Superior Electromagnetic Interference Shielding. Chem. Eng. J..

[9] Malere C.P.R., Donati B., Eras N., Silva V.A., Lona L.F. (2022). Electromagnetic Evaluation of Radar Absorbing Materials Based on Conducting Polypyrrole and Organic–Inorganic Nanocomposite of Polypyrrole/Kaolinite. J. Appl. Polym. Sci..

[10] Wang Y., Gao X., Zhang L.J., Wu X.M., Wang Q.G., Luo C.Y., Wu G.L. (2019). Synthesis of Ti_3_C_2_/Fe_3_O_4_/PANI Hierarchical Architecture Composite as an Efficient Wide-band Electromagnetic Absorber. Appl. Surf. Sci..

[11] Zainuri M., Amalia L. (2017). The Microstructure Analysis of Barium M-Hexaferrite Particles Coated by Pani Conducting Material with In Situ Polymerization Process. IOP Conf. Ser. Mater. Sci. Eng..

[12] Rana K., Thakur P., Tomar M., Gupta V., Thakur A. (2018). Investigation of Cobalt Substituted M-type Barium Ferrite Synthesized Via Coprecipitation Method for Radar Absorbing Material in Ku-band (12~18 GHz). Ceram. Int..

[13] Shen Z.Y., Xing H.L., Zhu Y.T., Ji X.L., Liu Z.F., Wang L. (2017). Synthesis and Enhanced Microwave-Absorbing Properties of SnO_2_/α-Fe_2_O_3_@RGO Composites. J. Mater. Sci. Mater. Electron..

[14] Fisli A., Winatapura D.S., Sukirman E., Mustofa S., Adi W.A., Taryana Y. (2019). Iron Oxide/titania Composites for Radar Absorbing Material (RAM) Applications. Cerâmica.

[15] Wagner D.V., Dotsenko O.A., Zhuravlev V.A. (2019). Structure Magnetic Properties and Electromagnetic Response of Y-Type Hexaferrites and Hexaferrite-Based Composite Materials. Russ. Phys. J..

[16] Feng C., Liu X.G., Or S.W., Ho S.L. (2017). Exchange Coupling and Micowave Absorption in Core/shell Structured Hard/soft Ferrite-Based CoFe_2_O_4_/NiFe_2_O_4_ Nanocapsules. AIP Adv..

[17] Folgueras L.C., Alves M.A., Rezende M.C. (2010). Dielectric Properties of Microwave Absorbing Sheets Produced with Silicone and Polyaniline. Mater. Res..

[18] Liu Y., Lai J., Liu Y. (2021). Preparaion Characterization and Microwave Absorpttion Properties of Cobalt-Doped SrFe_12_O_19_ Nanoparticles. J. Nanoelectron. Optoelectron..

[19] Li J., He S., Shi K.Z., Wu Y., Hong Y., Wu W.J., Meng Q.X., Jia D.C., Zhou Z.X. (2018). Coexistence of Broad-Bandwidth and Strong Microwave Absorption in Co^2+^-Zr^4+^, co-doped Barium Ferrite Ceramics. Ceram. Int..

[20] Ghasemi A., Hossienpour A., Morisako A., Saatchi A., Salehi M. (2006). Electromagnetic properties and microwave absorbing characteristics of doped barium hexaferrite. J. Magn. Magn. Mater..

[21] Feng G.L., Zhou W.C., Deng H.W., Chen D., Qing Y.C., Wang C.H., Luo F., Zhu D.M., Huang Z.B., Zhou Y.Y. (2019). Co Substituted BaFe_12_O_19_ Ceramics with Enhanced Magnetic Resonance Behavior and Microwave Absorption Properties in 2.6~18 GHz. Ceram. Int..

[22] Huang W., Wang Y.J., Wei S.C., Liang Y., Wang B., Huang Y.W., Xu B.S. (2020). Fabrication and microwave absorption properties of myrica rubra-like Fe_3_O_4_@SnO_2_ core-shell material. Chin. J. Eng..

[23] Kholil M.A., Priyono P., Subagio A. (2022). Multiwalled Carbon Nanotubes and Zinc Oxide Using a High Energy Milling Method for Radar-absorbent. Mater. Res. Express.

[24] Denny Y.R., Trenggono A., Firmansyah T., Revaldi I., Taryana Y., Aritonang S. (2021). Effect of Filler Concentration and Time Sonication of ZnO Composite for Radar Absorbing Material Applications. Mater. Sci. Forum.

[25] Ge Y.Q., Li G.P., Jiang X.H., Waterhouse G.I.N., Zhang L.J., Zhang Z.M., Yu L.M. (2019). ZnFe_2_O_4_@Polypyrrole Nanocomposites as An Efficient Broadband Electromagnetic Wave Absorber at 2−40 GHz. Ceram. Int..

[26] Yan M.H., Zhang Y.F., Fang Y.H., Yu L.M., Liu Y., Zhao X.L. (2018). The Electromagnetic Properties and Microwave Absorbing Performance of Titanium Carbide Attached Single-walled Carbon Nanotubes. J. Mater. Sci. Mater. Electron..

[27] Zhang D.Q., Jia Y.X., Cheng J.Y., Chen S.M., Chai J.X., Yang X.Y., Wu Z.Y., Wang H., Zhang W.J., Zhao Z.L. (2018). High-performance Microwave Absorption Materials Based on MoS_2_-Graphene Isomorphic Hetero-Structures. J. Alloy. Compd..

[28] Zhao D., Yuan X.Y., Li B.B., Jiang F., Liu Y., Zhang J.Y., Niu C.M., Guo S.W. (2020). Silicon Carbide Nanowire Covered by Vertically Oriented Graphene for Enhanced Electromagnetic Wave Absorption Performance. Chem. Phys..

[29] Uddin A., Estevez D., Qin F.X., Peng H.X. (2020). Programmable microwire composites: From functional units to material design. J. Phys. D Appl. Phys..

[30] Zi Z.F., Sun Y.P., Zhu X.B., Yang Z.R., Dai J.M., Song W.H. (2008). Structural and magnetic properties of SrFe_12_O_19_ hexaferrite synthesized by a modified chemical co-precipitation method. J. Magn. Magn. Mater..

[31] Gu F.M., Pan W.W., Liu Q.F., Wang J.B. (2013). Electrospun magnetic SrFe_12_O_19_ nanofibres with improved hard magnetism. J. Phys. D Appl. Phys..

[32] Strümpler R., Glatz-Reichenbach J. (1999). Conducting polymer composites. J. Electroceramics.

[33] Gao M.Z., Li Z.Y., Sun W.F. (2022). Nonlinear Conductivity and Space Charge Characteristics of SiC/Silicone Rubber Nanocomposites. Polymers.

[34] Tanaka T., Montanari G.C., Mulhaupt R. (2004). Polymer Nanocomposites as Dielectrics and Electrical Insulation perspectives for Processing Technologies, Material Characterization and Future Applications. IEEE Trans. Dielectr. Electr. Insul..

[35] Vinod P., Desai B.M.A., Sarathi R., Kornhuber S. (2019). Investigation on the electrical, thermal and mechanical properties of silicone rubber nanocomposites. IEEE Trans. Dielectr. Electr. Insul..

[36] Song W., Fan Y.Z., Hua Y., Sun W.F. (2020). Magnetic and Dielectric Properties of Nano- and Micron-BiFeO_3_/LDPE Composites with Magnetization Treatments. Materials.

[37] Venkatesulu B., Thomas M.J. (2010). Corona Aging Studies on Silicone Rubber Nanocomposites. IEEE Trans. Dielectr. Electr. Insul..

[38] Tu Y.P., Zhou F.W., Cheng Y., Jiang H., Wang C., Bai F.J., Lin J. (2018). The Control Mechanism of Micron and Nano SiO_2_/epoxy Composite Coating on Surface Charge in Epoxy Resin. IEEE Trans. Dielectr. Electr. Insul..

[39] Zhang Y.Q., Yu P.L., Sun W.F., Wang X. (2021). Ameliorated Electrical-Tree Resistant Characteristics of UV-Initiated Cross-Linked Polyethylene Nanocomposites with Surface-Functionalized Nanosilica. Processes.

